# Preparation and Thermal Properties of Magnetic PW@CaCO_3_@Fe_3_O_4_ Phase-Change Microcapsules and Their Application to Textile Fabrics

**DOI:** 10.3390/molecules29174151

**Published:** 2024-08-31

**Authors:** Xiaolei Song, Congzhu Xu, Hong Wei, Yonggui Li, Runjun Sun, Chunxia Wang, Jie Dong, Xinqun Feng

**Affiliations:** 1 Faculty of Clothing and Design, Minjiang University, Fuzhou 350108, China; sxlyanc@126.com (X.S.); xu15005932521@163.com (C.X.); 2 College of Textile Science and Engineering, Xi’an Polytechnic University, Xi’an 710048, China; sunrunjun@xpu.edu.cn (R.S.); dongjie@xpu.edu.cn (J.D.); 3 Hangzhou Jingsha Information Technology Co., Ltd., Hangzhou 311200, China; dacooler@163.com; 4 School of Textile and Clothing, Yancheng Institute of Technology, Yancheng 224051, China; cxwang@mail.dhu.edu.cn; 5 College of Fashion and Design, Donghua University, Shanghai 201620, China; fly6816_lele@163.com

**Keywords:** PW@CaCO_3_@Fe_3_O_4_, phase-change microcapsule, thermal property, magnetic, thermal regulation, fabrics

## Abstract

Multifunctional thermal regulation materials with good thermal properties, efficient magnetic performance, and satisfactory interface bonding on fabrics are highly desirable for protective fabrics, building winter protection materials, medical thermal regulation materials, and special-environment work clothing. Herein, a new class of magnetic phase-change PW@CaCO_3_@Fe_3_O_4_ microcapsules was successfully produced by controlling the content of magnetic Fe_3_O_4_ through a self-assembly method. The microstructure, chemical composition, phase-change behavior, and magnetic properties of the products were sequentially characterized and analyzed. The findings revealed that the obtained microcapsules possessed regular spherical structure with uniform size and excellent thermal properties. Furthermore, PW@CaCO_3_ with Fe_3_O_4_ (i.e., 8% mass fraction) showed the highest thermal regulation and magnetic properties and reached an enthalpy value of 94.25 J·g^−1^, which is clearly superior to the value of 77.51 J·g^−1^ for PW@CaCO_3_ microcapsules. At the same time, the encapsulation efficiency of 38.7% and saturation magnetization of 2.50 emu·g^−1^ were the best among the four given samples. Therefore, the good paramagnetic feature had a significant synergistic effect on the thermal properties of the PW@CaCO_3_ microcapsules under study. More importantly, multifunctional fabrics loaded with PW@CaCO_3_@Fe_3_O_4_ microcapsules still showed an enthalpy value of 25.81 J·g^−1^ after several washes and have the potential to be used widely in the field of temperature control. The thermal regulation fabrics in this study exhibited excellent thermal properties and fastness, which contribute to their practical applications in advancing multifunctional textiles and high-technology modern fabrics.

## 1. Introduction

With the continuous development of science and technology, multifunctional, intelligent textiles have gradually attracted widespread attention in recent decades in response to many studies of aspects such as heat, electricity, and mechanical forces; these textiles are achieving more diversified applications in the textile, building decoration, and medical protection fields [1,2,3]. In particularly, multifunctional, intelligent textiles are applied in magnetic thermal regulation fabrics due to the contribution of magnetic materials and phase-change microcapsule (PCM) materials. Magnetic PCM materials not only have the ability to regulate temperature but also possess characteristics such as magnetic responsiveness, energy efficiency, and multifunctionality. Consequently, magnetic PCMs have become one of the hotspots in intelligent textile research. The core of thermal regulation fabrics lies in their inclusion of PCMs, which are functional materials capable of storing and releasing thermal energy [4,5]. PCMs can regulate the surrounding environment’s temperature by absorbing or releasing heat within a specific temperature range. Their high thermal storage density permits a considerable amount of heat to be contained within a confined space. Moreover, the phase change occurs independently, without requiring any external energy input, resulting in substantial energy savings. Therefore, PCMs have been widely applied in various fields, including solar energy utilization, building insulation, functional textiles, medical devices, electronic products, and food refrigeration.

Conventional PCMs typically exhibit low thermal conductivity and are prone to leakage during phase transition [6]. Consequently, these drawbacks have restricted their practical applications in scope and depth. To overcome these challenges, researchers have recently enhanced the thermal conductivity and stability of PCMs by either coating them with inorganic shell materials or incorporating high-thermal-conductivity nanoparticles into PCMs. Zhao and his team members selected n-octadecane as the phase-change core material and crosslinked polystyrene as the shell material to prepare PCMs through suspension polymerization. Moreover, silicon carbide nanoparticles were used to modify the shell material of the microcapsules to improve their thermal conductivity and photothermal conversion efficiency [7,8,9,10]. The results indicated that compared with the undoped microcapsule samples, the samples with added silicon carbide nanoparticles exhibited enhanced thermal performance, conductivity, and photothermal conversion efficiency. Especially, the photothermal conversion efficiency reached up to 54.91% in the study. Yu et al. [11] selected solar salt (a binary eutectic salt with a mass ratio of NaNO_3_:KNO_3_ of 3:2) as the PCM and silicon dioxide (SiO_2_) as the shell material. However, their study did not focus on the effect of magnetic ferrite on phase-change microcapsules. By introducing high thermal conductivity and superparamagnetic carbon nanotube and iron oxide nanoparticles (CNTs-Fe_3_O_4_) composite material as the enhancement phase, they prepared multifunctional magnetic PCMs. The results indicated that the encapsulation of SiO_2_ and the introduction of CNTs-Fe_3_O_4_ increased the thermal conductivity of the microcapsules by approximately 53.4%. Furthermore, the microcapsules exhibited superparamagnetic properties and photothermal conversion characteristics, suggesting remarkable potential for applications in energy collection, conversion, and storage. Cai et al. [12] used paraffin was (PW) as the phase-change core material and Fe_3_O_4_ nanoparticles modified with melamine–formaldehyde resin as the shell material to prepare multifunctional microcapsules. Although the addition of magnetic Fe_3_O_4_ nanoparticles can generate a significant synergistic effect on the thermal properties of the microcapsules, but their research did not explore its application in the textile protective field. Analysis revealed that these PCMs possess not only infrared stealth and microwave absorption effects but also temperature regulation and electromagnetic radiation absorption properties. Therefore, we will continue to explore the synergistic function of Fe_3_O_4_ with PW@CaCO_3_ microcapsules’ thermal properties and their wide application prospects in the fields of fabric production for national defense and civilian construction.

CaCO_3_ is highly favored due to its widespread availability, low cost, high mechanical strength, stable performance, and environmental friendliness. As the shell material, CaCO_3_ is easy to process into a microcapsule structure with controllable size and wide compatibility, making it excellent for exploratory work on phase-change microcapsules. Meanwhile, Fe_3_O_4_ nanoparticles are extensively used in the study of modified PCMs because of their high thermal conductivity and size effect advantages [13]. Therefore, in this paper, PW was first selected as the core material, and CaCO_3_ doped with Fe_3_O_4_ nanoparticles was used as the hybrid shell material. Using the self-assembly method, magnetic phase-change microcapsules with different quantities of Fe_3_O_4_ nanoparticles were prepared. Subsequently, the dipping method was employed for the post-treatment of pure cotton fabrics, ensuring that their surfaces were uniformly covered with microcapsules, thereby endowing them with excellent temperature-regulating properties. Finally, the microstructure, chemical composition, phase-change behavior, and magnetic properties of the microcapsules and the surface morphology and temperature-regulating properties of the treated fabrics were systematically characterized and analyzed. Through these studies, new inspirations and ideas for the development of multifunctional thermal regulation fabrics are provided.

## 2. Results

### 2.1. Illustration of the Preparation Process

**Figure 1:**
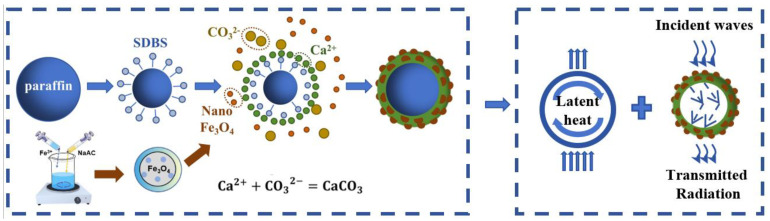
Schematic representation of the preparation of PW@CaCO_3_@Fe_3_O_4_ PCMs.

Figure 1 presents the preparation mechanism of PW@CaCO_3_@Fe_3_O_4_ PCMs, including the preprocessing of Fe_3_O_4_ nanoparticles and the self-assembly of PCMs. First, magnetic Fe_3_O_4_ nanoparticles were successfully prepared by our previous research [14]. Second, PW was dissolved with 15% mass percentage of sodium dodecyl benzene sulfonate (SDBS), which was extended toward the PW particles as follows. Subsequently, some CaCl_2_ solution was slowly added, reacting with the hydrophilic groups to form a uniform complex. Next, Na_2_CO_3_ solution and prefinished Fe_3_O_4_ nanoparticles were gradually introduced. The Ca^2+^ then reacted with CO_3_^2−^ to encapsulate the PW particles uniformly, forming the hybrid shell material [15,16]. Ultimately, the PW@CaCO_3_@Fe_3_O_4_ PCMs were prepared for fine shaping, together with predesign thermal regulation and electromagnetic radiation protection, as shown in Figure 1.

### 2.2. Surface Morphology Analysis of Microcapsules

**Figure 2:**
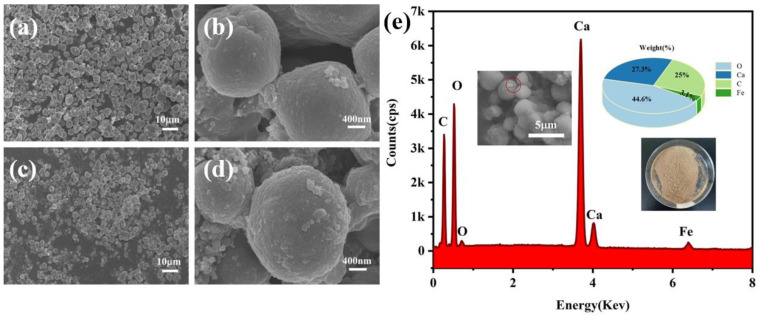
SEM images: (**a**,**b**) PW@CaCO_3_ and (**c**,**d**) PW@CaCO_3_@Fe_3_O_4_ PCMs at different magnifications; (**e**) EDS energy spectrum of PW@CaCO_3_@Fe_3_O_4_ PCMs.

The surface morphology of the microcapsules is depicted in Figure 2. Figure 2a,b display the shape of the PW@CaCO_3_ PCMs, which appear as regular spheroids with clear outlines and even distribution. It can be seen that the particle size of the microcapsules ranges from approximately 2 μm to 4 μm. Furthermore, Figure 2c,d reveals the scanning electron microscopy (SEM) images of the microcapsules containing 0.8 g of Fe_3_O_4_ nanoparticles. No obvious Fe_3_O_4_ nanoparticles are present on the surface of the microcapsules, suggesting that the incorporation of Fe_3_O_4_ nanoparticles does not affect the original morphology of the PW@CaCO_3_ phase microcapsules [17,18]. The obtained PW@CaCO_3_@Fe_3_O_4_ microcapsules have a larger particle size than the pure microcapsules. Figure 2e illustrates the energy-dispersive spectroscopy (EDS) results of the red-marked field of PW@CaCO_3_@Fe_3_O_4_ (8%) PCMs. As observed, the surface of the microcapsule is mostly made of the elements O, Ca, C, and Fe, illustrating that the test capsule’s shell material is composed of CaCO_3_ and Fe_3_O_4_ through the characteristic peak [19].

### 2.3. Analysis of the Chemical Structure of the Microcapsules

**Figure 3:**
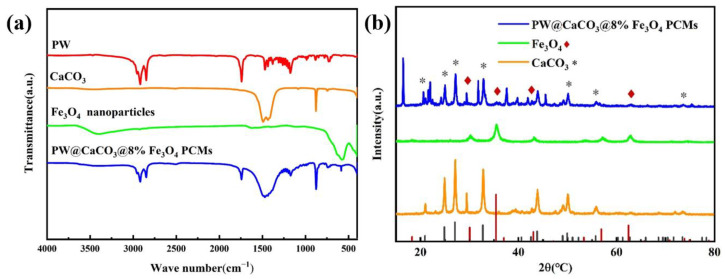
(**a**) FTIR patterns of PW, CaCO_3_, Fe_3_O_4_, and PW@CaCO_3_@Fe_3_O_4_ PCMs and (**b**) XRD patterns of CaCO_3_, Fe_3_O_4_, and PW@CaCO_3_@Fe_3_O_4_ PCMs.

Fourier transform infrared (FTIR) spectroscopy analysis was performed to assess the chemical structure of PW, CaCO_3_, Fe_3_O_4_ nanoparticles, and composite microcapsules. In Figure 3a, the diffraction curve of pure PW shows characteristic peaks at 2915 and 2848 cm^−1^, corresponding to the C-H stretching vibrations of the -CH_3_ and -CH_2_ groups, respectively. The distinctive peaks for CaCO_3_ appear at 708 and 875 cm^−1^, associated with the in-plane and out-of-plane bending vibrations of CO_3_^2−^, respectively. However, the peak at 1475 cm^−1^ is attributed to the asymmetric stretching vibrations of CO_3_^2−^ in CaCO_3_ [20]. The absorption peak at 575 cm^−1^ is linked to the Fe-O group obtained from the Fe_3_O_4_ crystal structure. The PW@CaCO_3_@Fe_3_O_4_ PCM curve exhibits the characteristic absorption peaks without the appearance of any new peak. The C-H stretching vibration peaks of PW, and the characteristic peaks of CaCO_3_ are still presented, together with the Fe-O absorption peak of Fe_3_O_4_. The characteristic absorption peaks of PW, CaCO_3_, and Fe_3_O_4_ can be simultaneously observed in the spectrum of PW@CaCO_3_@Fe_3_O_4_ PCM, indicating that these individual components have been successfully embedded into the microcapsule structure while retaining their respective chemical characteristics [21]. This observation confirms the successful synthesis of the microcapsules through a self-assembly method. This observation also suggests that the three components physically combine without any unnecessary chemical reaction in the preparation. Furthermore, Figure 3b shows the crystal structures of CaCO_3_, Fe_3_O_4_ nanoparticles, and the magnetic PW@CaCO_3_@Fe_3_O_4_ PCMs. The diffraction peaks of the obtained microcapsules at 2θ angles of 20.91°, 24.87°, 27.03°, 32.71°, 49.92°, 55.73°, and 73.45°, correspond to the diffraction peaks of CaCO_3_’s crystal planes (002), (100), (101), (102), (104), (202), and (212), respectively [22]. Additionally, the 2θ angles of 30.35°, 35.39°, 43.01°, and 62.46° are consistent with the characteristic diffraction peaks of the Fe_3_O_4_ nanoparticles, aligning with the crystal planes (220), (311), (400), and (440), respectively [23]. Consequently, the diffraction pattern of the PW@CaCO_3_@Fe_3_O_4_ PCMs exhibits the characteristic crystal planes of both CaCO_3_ and Fe_3_O_4_ sections, which is highly consistent with the primary study [14].

### 2.4. Analysis of the Thermal Properties of the Microcapsules

**Figure 4:**
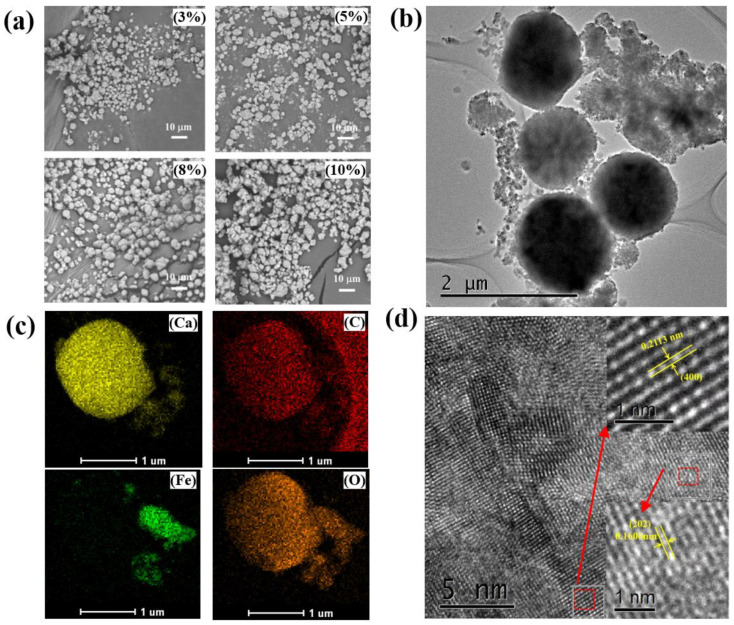
(**a**) SEM images of PW@CaCO_3_ microcapsules with Fe_3_O_4_ content of 3%, 5%, 8%, and 10%; (**b**) TEM, (**c**) mapping, and (**d**) HRTEM of PW@CaCO_3_ microcapsules with 8% Fe_3_O_4_.

**Figure 5:**
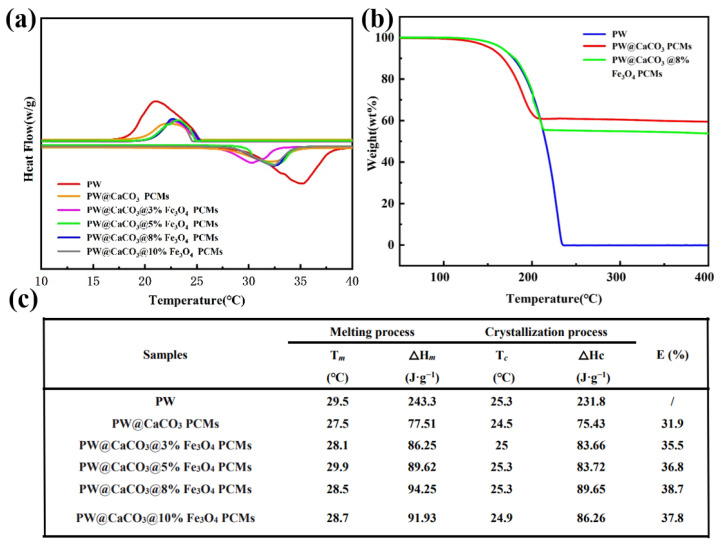
(**a**) DSC and (**b**) TG curves of PW and microcapsules; (**c**) DSC data of microcapsules.

The morphology of the PW@CaCO_3_ magnetic PCMs with different mass fractions of Fe_3_O_4_ is illustrated in Figure 4a. The incorporation of Fe_3_O_4_ nanoparticles does not affect the original spherical structure of the PW@CaCO_3_ PCMs, showing the spherical shape and fine distribution. Notably, microcapsules containing 8% Fe_3_O_4_ in Figure 4b show the optimum state with uniform dispersion, with an average particle size around 2 μm. Furthermore, the core-shell structure of the obtained capsules are obvious, with a number of tiny particles around the shell edge. The combination of the PW@CaCO_3_ microcapsules and Fe_3_O_4_@ nanoparticles shows higher binding action in the process of self-assembly. However, the visual element mapping is described as Figure 4c. There are uniformly dispersed Ca, C, and O elements in two-dimensional composites, containing bit Fe elements evenly distributed on the surface and more concentrated on the edge of the sphere microcapsule, illustrating the test capsule’s shell material is composed of CaCO_3_ and Fe_3_O_4_ through the characteristic. We can see that the lattice spacing of 0.2132 nm corresponds to CaCO_3_’s crystal plane (202), and 0.1606 nm corresponds to Fe_3_O_4_’s crystal plane (400), respectively. As observed, the results further illustrate the role of the co-existence of CaCO_3_ and Fe_3_O_4_ in the forming process of shell [24,25]. However, the differential scanning calorimetry (DSC) curves and data of the samples are shown in Figure 5a,c, which provides detailed information on the enthalpy values, phase-change temperatures, and encapsulation rates of the microcapsule samples. The enthalpy value of the PW@CaCO_3_ PCMs is 77.51 J·g^−1^. With the addition of Fe_3_O_4_ nanoparticles, the curves of enthalpy initially increase to 94.25 J·g^−1^ of 0.8 g Fe_3_O_4_ and then decrease to 91.93 J·g^−1^ with the constantly increasing addition [26]. Li have successfully prepared Fe_3_O_4_ nanocomposites with flower-like and chain-like shapes. They showed higher coercive force values than our samples’, which is not suitable for soft magnetic properties required low coercivity for protective fields [27]. Shao’s team prepared PCMs with equivalent Fe_3_O_4_ nanoparticles exhibited an enthalpy value of 85 J·g^−1^ as measured by the seed microsuspension polymerization method; this value is lower than our sample’s 91.93 J·g^−1^ [28]. The result shows that the heat storage performance of our study is better, leading to a broader application prospect. Figure 5b illustrates the thermal degradation behaviors of PW, PW@CaCO_3_, and PW@CaCO_3_@Fe_3_O_4_ PCMs. According to the thermal gravimetric (TG) curves, PW starts to decompose at around 120 °C, and its maximum mass loss occurs near 230 °C. Similarly, PW@CaCO_3_ and PW@CaCO_3_@Fe_3_O_4_ PCMs begin to decompose at 120 °C. The PW@CaCO_3_ PCMs experiences a 62% weight loss at 210 °C, whereas the PW@CaCO_3_@Fe_3_O_4_ PCMs encounters a 55% weight loss at 220 °C. As the temperature reaches 400 °C, no further weight changes are observed. At this stage, the core material PW has completely vaporized, leaving only the Fe_3_O_4_ nanoparticles and the CaCO_3_ shell material [29].

### 2.5. Analysis of the Magnetic Properties of the PCMs

**Figure 6:**
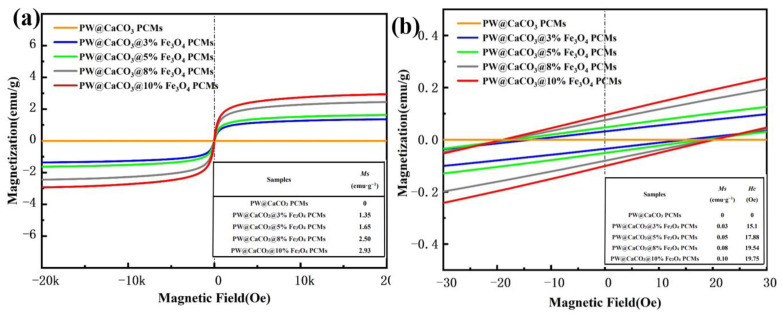
Hysteresis loops (**a**) and magnified hysteresis loops (**b**) of the PW@CaCO_3_@Fe_3_O_4_ PCMs.

The magnetic properties of the PW@CaCO_3_ PCMs with varying Fe_3_O_4_ mass fractions are analyzed using a vibrating sample magnetometer (VSM), as illustrated by the hysteresis loops in Figure 6a,b. The magnetization curve is also called the hysteresis curve, which reflects the relationship between the magnetic induction intensity of the magnetic material and the magnetic field intensity. We can see that each sample exhibits an S-shaped hysteresis loop, indicating low coercivity and remanent magnetization under an external magnetic field with fine ferromagnetism [30,31,32,33]. Specifically, with a 3% mass fraction of Fe_3_O_4_ nanoparticles, the magnetic microcapsules achieve a magnetization saturation strength of just 1.35 emu·g^−1^. This value rises to 2.93 emu·g^−1^ when the Fe_3_O_4_ nanoparticles mass fraction is increased to 10%. However, the coercivity values are similar and low than 20 Oe, illustrating the soft magnetic properties are close and stable among the four samples, which are shown in the interior tables as follows [34]. With the rise of the soft magnetic materials, it may be used to develop smart wearable textiles with specific functions, for example, magnetic shielding clothing to protect the wearer from electromagnetic radiation or functional magnetic therapy textiles to improve the wearer’s blood circulation and relieve pain [35].

### 2.6. Performance Analysis of the Thermal Regulation Fabrics

**Figure 7:**
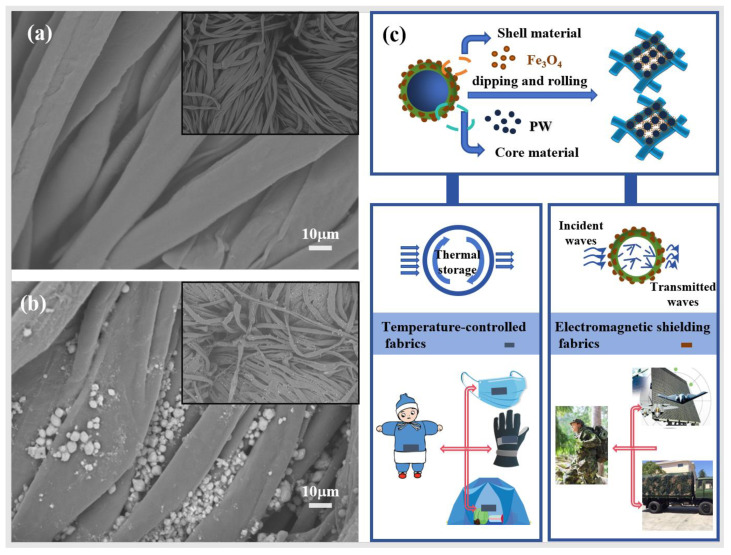
(**a**) SEM image of cotton fabric, (**b**) SEM image of thermal regulation fabric, and (**c**) scenario diagram of the preparation and application of thermal regulation fabric.

Figure 7 shows the surface morphology of the fabrics as well as the preparation principle and application scenario schematic diagram. Figure 7a,b show the SEM images of pure cotton fabric and thermal regulation fabric, respectively. The PCMs are evenly distributed on the fiber surface and inside the fibrous septum, confirming that the microcapsules have been successfully loaded on the fabrics through the dip coating method [36,37,38,39]. In the process, the finishing liquid enters the structural gap of the fabric through the pressure of the counter-rotating rolling mills [40,41]. Consequently, the microcapsules are spread unevenly across the fabric surface, leading to low concentrations in certain regions. Figure 7c describes the preparation principle and application scenario of the functional fabrics, including thermal regulation products and electromagnetic shielding fabrics.

**Figure 8:**
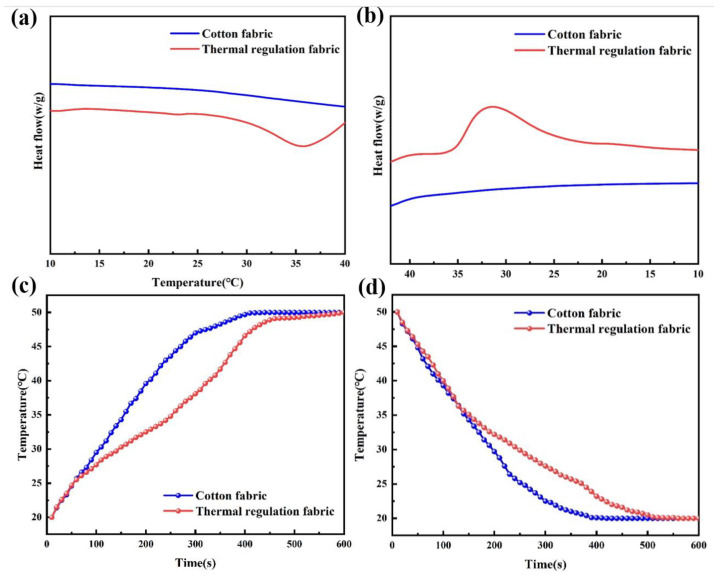
DSC curves of the fabrics during a temperature rise (**a**) and drop (**b**); time–temperature point plots of the fabrics during a temperature rise (**c**) and drop (**d**).

Figure 8a,b illustrate the DSC curves of the above fabrics. Pure cotton fabric shows very low thermal properties. Following the dipping treatment, the enthalpy values of the thermal regulation fabric for melting and crystallization are 25.81 J·g^−1^ and 25.79 J·g^−1^, respectively. Additionally, the phase transition temperature spans from 15 °C to 39 °C. Figure 8c illustrates the time–temperature curve for pure cotton fabric and thermal regulation fabric at temperature rise. Initially, both fabrics are heated from room temperature to 50 °C. Notably, as the regulation fabric approaches its phase transition temperature, its heating rate diminishes, demonstrating its ability to regulate temperature [42,43,44]. By the 9-min mark, both fabrics have exceeded 38 °C, indicating that the thermal regulation fabric can maintain its phase-change performance for approximately 8 min during heating. Figure 8d illustrates the time–temperature curve of the pure cotton fabric and the thermal regulation fabric at temperature drop. Both fabrics cool from 50 °C to approximately 20 °C simultaneously. In the initial 2 min, the cooling rates of both fabrics are nearly identical, and both drop below 37 °C at 2 min. Subsequently, the cooling rate of the fabric gradually decelerates until 9 min, at which point the temperatures of both fabrics decrease to about 20 °C, indicating that the thermal regulation fabric exhibits certain phase transition performance during cooling and can maintain this state for approximately 7 min.

The softness of the fabrics was evaluated using a fabric stiffness tester, measuring bending length and flexural rigidity to assess its soft property. The bending rigidity substantially increases from 57.696 mg·cm to 75.307 mg·cm, illustrating the decline in softness of the dip-rolled fabric in Table 1. However, the bending lengths are similar due to the finishing solution with the microcapsules and appropriate additives, which establishes fine linking at the interface of the functional fabrics [45,46]. Consequently, the mechanical properties of the fabric have been notably enhanced after the dipping treatment.

Table 2 shows the melting and crystallization temperatures along with the enthalpy changes of the fabrics after several wash cycles [47]. While the phase transition temperatures remain consistent, the enthalpy changes considerably. Specifically, the thermal enthalpy of the thermal regulation fabric decreases after several times of washing. The decline is due to the partial removal of microcapsules from the fabric. After three and five times of washing, the fabric retains 88.11% and 82.06% of its melting enthalpy, respectively. However, the decline degree is remarkably slow when the number of washes is increased from three to five. Hence, the stability of the dipping fabrics is gradually achieved when it is washed five times. However, Chen have studied the effect of ten times washing on the fastness and heat storage properties of the microcapsules. The residue rate was higher than our samples, but the thermal performance is not as good as our samples’ enthalpy value of 21.18 J·g^−1^ [48].

## 3. Materials and Methods

### 3.1. Materials

PW (melting point: 28 °C) was purchased from Dongguan Donglin New Materials Co., Ltd. (Dongguan, China). Anhydrous calcium chloride (CaCl_2_), SDBS, and polyethylene glycol 400 (PEG-400) were obtained from Shanghai Macklin Biochemical Technology Co., Ltd. (Shanghai, China). Sodium carbonate (Na_2_CO_3_) and anhydrous ethanol were acquired from Xilong Scientific Co., Ltd. (Guangzhou, China). Water-based polyurethane adhesive was purchased from Anhui Dawei Huatai Technology New Material Co., Ltd. (Hefei, China). Deionized water was used during the experiments.

### 3.2. Preparation of PW@CaCO_3_@Fe_3_O_4_ PCMs

First, spherical Fe_3_O_4_ nanoparticles with uniform structures were prepared using a repeated synthesis method similar to that described in our previous study. Then, 1.5 g of SDBS was dissolved into 100 mL of deionized water and stirred magnetically for 20 min to ensure a homogeneous mixture. At the same time, 10 g of PW was poured into a three-neck flask and heated at 40 °C water bath until completely melted. Subsequently, the mixed SDBS solution was dropwise added to the three-neck flask and stir mechanically at 1000 RPM for 30 min to obtain the oil phase solution. Second, 10 g of CaCl_2_ was dissolved in 100 mL of deionized water and magnetically stirred for 20 min. Simultaneously, Na_2_CO_3_ powder was conducted to 100 mL of deionized water with varying mass fractions of Fe_3_O_4_ nanoparticles at 50 °C for 3 h. Third, the CaCl_2_ solution was gradually introduced into the three-neck flask while stirring at 1000 RPM for 3 h. Then, the Na_2_CO_3_ solution was introduced slowly at 800 RPM for another 3 h. Finally, the PW@CaCO_3_@Fe_3_O_4_ PCMs were washed thoroughly with deionized water, followed by drainage and drying in the following experiments.

### 3.3. Preparation of Thermal Regulation Fabrics

First, the prepared cotton fabric was immersed in a sodium hydroxide (NaOH) solution at 70 °C for 60 min for alkali treatment and then dried. Second, the previously mentioned microcapsules were measured while adding deionized water and stirred mechanically for 30 min to achieve a uniformly mixed impregnation finishing solution. Third, the fabric was placed into the above solution, and a liquor ratio of 1:50 was maintained for 3 h of ultrasonic assistance. Then, a rolling mill of double dipping and double rolling was conducted to produce the finished fabric. Finally, all the fabric samples were prebaked at 80 °C for 10 min, baked at 120 °C for 3 min, and allowed to dry naturally to obtain the thermal regulation fabrics for further study.

### 3.4. Characterizations

The surface morphology of the microcapsules and fabrics was examined using field emission SEM. Elemental analysis of the magnetic phase-change microcapsules was performed using X-ray spectroscopy (EDS). The crystal structures of CaCO_3_, Fe_3_O_4_ nanoparticles, and the microcapsules were analyzed by X-ray diffraction (XRD) within a 2θ scan range of 10° to 80° at 40 kV and 40 mA. FTIR Spectroscopy was utilized to investigate the chemical functional group composition of PW, CaCO_3_, Fe_3_O_4_, and the microcapsules, covering a range from 4000 cm^−1^ to 400 cm^−1^. The melting and crystallization temperatures, along with the enthalpies of PW, microcapsules, and fabrics were determined using DSC. These measurements were conducted from 0 °C to 50 °C at a heating rate of 10 °C/min under a nitrogen atmosphere. The thermal stability of PW and the microcapsules was assessed via TG analysis by heating the samples from room temperature to 400 °C in an aluminum crucible at a rate of 10 °C/min. The magnetic properties of the PW@CaCO_3_@Fe_3_O_4_ PCMs were characterized with a VSM over a range of −20 KOe to 20 KOe at room temperature. The thermal regulation performance of the fabrics was evaluated using a flat temperature-retaining instrument, and data were recorded through an infrared thermographic camera to generate heating and cooling time–temperature curves. Finally, the softness of the obtained fabrics was tested using a fabric stiffness tester.

## 4. Conclusions

In this comprehensive study, magnetic PCMs of PW@CaCO_3_@Fe_3_O_4_ were produced from varying Fe_3_O_4_ fractions serving as hybrid shell materials through a self-assembly method. The main findings of the study regarding the synergistic function of Fe_3_O_4_ with PW@CaCO_3_ microcapsules’ thermal properties and the broader application prospects of thermal regulation fabrics are as follows.

(1) The appearance, phase transformation, and magnetic properties of the obtained microcapsules were assessed to determine their thermal performance. The XRD and FTIR results confirmed the successful preparation of the target magnetic microcapsules. The SEM and TEM images showed that the microcapsules were spherical and uniformly distributed, measuring approximately 2 μm in diameter. When the Fe_3_O_4_ nanoparticle mass fraction was 8%, the microcapsules exhibited a melting enthalpy of 94.25 J·g^−1^ and a saturation magnetization of 2.50 emu·g^−1^ according to DSC and VSM analyses.
(2) Cotton fabric, serving as the base material, was impregnated and finished with a solution comprising an adhesive and microcapsules to create functional thermal regulation fabrics. The structure and properties of the obtained fabrics were measured and analyzed in succession. The impregnated fabrics demonstrated good appearance and softness properties, together with thermal performance.
(3) Under optimal processing conditions, the tempering fabric’s phase-change enthalpy reached 25.81 J·g^−1^, maintaining phase-change behavior for 8 min during heating. Additionally, the enthalpy retention rate was 82.06% after five washing cycles.

## Figures and Tables

**Table 1:** Mechanical properties of fabrics.

Samples	Bending Length (cm)	Bending Rigidity (mg·cm)
Cotton fabric	1.56	57.696
Thermal regulation fabric	1.57	75.301

**Table 2:** Phase-change characteristics of fabrics after different wash cycled.

Washing Times	Melting Process		Crystallization Process	
	T_m_ (°C)	△H_m_ (J·g^−1^)	T_c_ (°C)	△H_c_ (J·g^−1^)
0	31.1	25.81	27.9	25.79
3	31.2	22.74	27.7	21.63
5	31.1	21.18	27.7	20.65

## Data Availability

Data are contained within the article.
